# Impulsive choice in hippocampal but not orbitofrontal cortex-lesioned rats on a nonspatial decision-making maze task

**DOI:** 10.1111/j.1460-9568.2009.06837.x

**Published:** 2009-08

**Authors:** T Y Mariano, D M Bannerman, S B McHugh, T J Preston, P H Rudebeck, S R Rudebeck, J N P Rawlins, M E Walton, M F S Rushworth, M G Baxter, T G Campbell

**Affiliations:** Department of Experimental Psychology, University of OxfordSouth Parks Road, Oxford, OX1 3UD, UK

**Keywords:** delay discounting, reversal, spatial, T-maze, water maze

## Abstract

Orbitofrontal cortical (OFC) and hippocampal (HPC) lesions in primates and rodents have been associated with impulsive behaviour. We showed previously that OFC- or HPC-lesioned rats chose the immediate low-reward (LR) option in preference to the delayed high-reward (HR) option, where LR and HR were associated with different spatial responses in a uniform grey T-maze. We now report that on a novel nonspatial T-maze task in which the HR and LR options are associated with patterned goal arms (black-and-white stripes vs. gray), OFC-lesioned rats did not show impulsive behaviour, choosing the delayed HR option, and were indistinguishable from controls. In contrast, HPC-lesioned rats exhibited impulsive choice in the nonspatial decision-making task, although they chose the HR option on the majority of trials when there was a 10-s delay associated with both goal arms. The previously reported impairment in OFC-lesioned rats on the spatial version of the intertemporal choice task is unlikely to reflect a general problem with spatial learning, because OFC lesions were without effect on acquisition of the standard reference memory water-maze task and spatial working memory performance (nonmatching-to-place) on the T-maze. The differential effect of OFC lesions on the two versions of the intertemporal choice task may be explained instead in terms of the putative role of OFC in using associative information to represent expected outcomes and generate predictions. The impulsivity in HPC-lesioned rats may reflect impaired temporal information processing, and emphasizes a role for the hippocampus beyond the spatial domain.

## Introduction

Orbitofrontal cortex (OFC) damage in humans is commonly associated with impulsive behaviour (Cummings, 1993). Impulsivity has also been observed in rodents with OFC lesions (Kheramin *et al.*, 2002; Mobini *et al.*, 2002). For example, we have recently shown using a spatial intertemporal choice task that OFC lesions increase rats’ preferences for the low-reward (LR) arm of a T-maze in which reinforcement is immediate, compared to the high-reward (HR) arm in which food is only available after a delay (Rudebeck *et al.*, 2006). It has therefore been suggested that the OFC is essential for choosing delayed rewards over immediate rewards (Mobini *et al.*, 2002; but see also Winstanley *et al.*, 2004). Hippocampal (HPC) lesions also cause impulsive behaviour (Rawlins *et al.*, 1985; Cheung & Cardinal, 2005), and hippocampal-lesioned rats also display an increased preference for the immediate LR option on the same spatial intertemporal choice T-maze task (McHugh *et al.*, 2008).

**Fig. 1:**
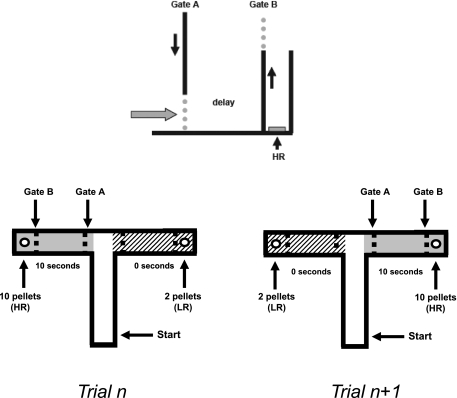
Experimental apparatus for the ‘cued/nonspatial’ delay-based cost–benefit decision-making task. Rats were placed in the start arm of the T-maze and allowed to choose between the two goal arms, one painted gray and the other painted with black and white stripes. The right–left orientation of the gray and black-and-white goal arms varied according to a pseudorandom sequence. When rats entered one of the goal arms, gate A was immediately closed, keeping the rat in that goal arm. Gate B was then opened after the required delay period. The high reward (HR) was always associated with a particular colour of goal arm.

To test the generality of OFC and HPC involvement in intertemporal choice, we trained rats on a novel, nonspatial T-maze task in which the delayed HR and immediate LR options were associated with patterned goal arms (black and white stripes vs. gray), whose right–left orientation varied from trial to trial (Fig. 1). If an intact OFC is required for suppressing the impulsive choice of immediate reward over a delayed larger reward then OFC lesions should impair performance on this task just as they did in previous experiments (Rudebeck *et al.*, 2006). A similar prediction can be made for the effects of HPC lesions (McHugh *et al.*, 2008). Importantly, an impairment in the nonspatial intertemporal choice task following lesions of the HPC would indicate that the involvement of the HPC in decision making is not limited to settings in which the choice is between spatial locations. The rats in this experiment were also tested in spatial working memory (T-maze alternation), spatial learning and reversal (repeated acquisition) learning in a Morris water-maze, and food neophobia, to provide additional measures of the functional effectiveness of HPC and OFC lesions for interpretation of the data obtained in the intertemporal choice task.

## Materials and methods

### Subjects

Thirty-six experimentally naïve male Lister-Hooded rats (Harlan, OLAC, Bicester, UK), approximately 2 months old at the start of behavioural testing, served as subjects in all of these experiments. Animals were housed in groups of two or three under a 12-h light–dark cycle (lights on 07.00–19.00 h) in a temperature- and humidity-controlled room. Unless otherwise stated, rats had access to food and water *ad libitum* in their home cages. These experiments were conducted under the authority of UK Home Office project and personal licenses held by the authors.

### Experiment 1: nonspatial, cued, delay cost–benefit decision making

#### Apparatus

Testing was conducted in an enclosed wooden T-maze (arms 61 × 10 × 39 cm; Fig. 1). The start arm, which was unpainted (natural wood colour), led to the two goal arms which were painted in different colours: one with alternate black and white stripes (approximately 2 cm wide) and the other uniform grey. The black-and-white or grey colour covered the entire walls and floor of the goal arm from the entrance to the back wall behind the food well. Within each goal arm were two 50-cm-high guillotine doors (also painted grey or with black and white stripes as appropriate), one 5 cm from the start of the goal arm (gate A) and one 10 cm from the end wall of the goal arm (gate B). These doors could be independently moved so as to restrict or allow access to various portions of the goal arm. The doors were used to contain the rat in the goal arm in order to be able to impose a delay between making a choice and receiving the reward. A metal food well (3 cm in diameter) was located 5 cm from the end wall of each goal arm. The two goal arms were interchangeable, and they could be removed from the maze and have their spatial locations swapped (Fig. 1).

#### Preoperative training

Rats were maintained on a restricted feeding schedule at 85% of their free-feeding body weight. They were then fully habituated to the maze. During this stage of pre-training, unpainted (natural wood colour) goal arms were used. Rats were initially habituated to the maze in groups of two or three, with food (45-mg Noyes pellets; Sandown Scientific, UK) freely available in both goal arms. They were then made to run individually for food in each goal arm by preventing access to the alternate goal arm using gate A. Once all the animals were running freely and readily consuming the food rewards, training on the reward discrimination task began.

**Fig. 4:**
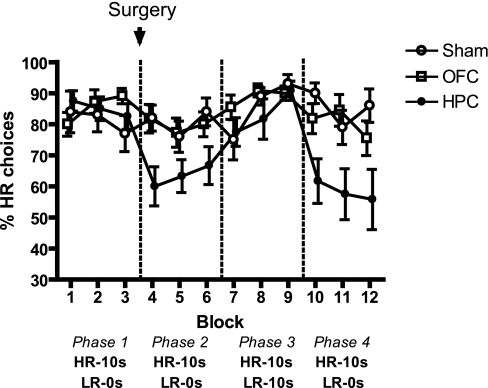
HPC but not OFC lesions resulted in impulsive choice on the ‘cued/nonspatial’ delay-based cost–benefit decision-making task. Mean (±SEM) percentage of trials on which rats chose the delayed HR option. During phase 1 (preoperative testing), all animals chose the delayed HR option on the majority of trials. After surgery (phase 2), both OFC-lesioned rats (white squares) and sham-operated controls (white circles) continued to choose the delayed HR option. In contrast, the HPC-lesioned rats (black circles) displayed impulsive choice behaviour and chose the immediate LR option more frequently than did the controls. However, when an equivalent delay was present in both the HR and LR arms (phase 3), the HPC-lesioned rats returned to choosing the HR option on the majority of trials. Reintroduction of the original training contingencies (delayed HR vs. immediate LR; phase 4) reinstated the deficit in the HPC-lesioned rats, which reverted to choosing the immediate LR goal arm with increased frequency.

Rats were first trained to choose between a LR arm which contained two food pellets and a HR arm which contained 10 pellets. For half of the rats, the HR was associated with the black-and-white striped goal arm and the LR with the grey goal arm. For the remaining rats, the allocations were reversed. There were no delays present during this stage of training and rats had immediate access to either reward. On each day of testing, rats first received two forced trials at the start of each training session (by closing the appropriate gate A), one to the HR arm and one to the LR arm (the order of the forced trials was according to a pseudorandom sequence). The rats then received five free-choice trials and their choices were recorded. The left–right orientation of the HR and LR arms was varied according to a pseudorandom sequence with no more than two consecutive trials with the arms in the same configuration. The number of trials in which the HR was on the left or the right was balanced across two consecutive test sessions (i.e. across each block of 10 choice trials). Training in this zero delay condition continued for 12 days (60 trials) until all the animals were choosing the HR on > 80% of trials. At this point, a 5-s delay was introduced into the HR arm. The doors (gate B) at the end of both goal arms were now initially closed. If the rat chose the HR arm, gate B was kept shut and the rat was detained in the goal arm by closing gate A. After a delay of 5 s gate B was raised, allowing access to the HR. If the rat chose the LR, gate B was raised immediately and thus there was no additional delay to reinforcement. Each test session involved two forced trials and five choice trials. After three training sessions with a 5-s delay, the delay in the HR arm was further increased to 10 s. Rats then received six test sessions (two forced and five choice trials per session) with a 10-s delay to reinforcement in the HR arm, and with immediate access to the food in the LR arm. Choice trial data from two consecutive sessions were combined into a single block of 10 trials for analysis (Fig. 4; blocks 1–3; phase 1). The rats were then returned to *ad libitum* food and allocated to groups for surgery.

#### Surgery

After preoperative training on the delay task, subjects were allocated to one of three groups, counterbalanced with respect to preoperative performance and whether the HR was associated with the striped or grey goal arm. Rats received either bilateral lesions of the complete hippocampus (HPC; *n* = 12; McHugh *et al.*, 2008), bilateral lesions of the OFC (*n* = 12; Rudebeck *et al.*, 2006), or sham lesions (*n* = 12). The animals were anaesthetised with isoflurane (Animal Care, York, UK) and then placed in a stereotaxic frame with the head level (Kopf Instruments, Clark Electromedical, Reading, UK; or Stoelting Company, Illinois, USA). Excitotoxic lesions of the OFC were produced using quinolinic acid (0.09 M), and lesions of the HPC were made using *N*-methyl-d-aspartate (0.068 M), both of which were dissolved in phosphate buffer solution (pH 7.4; both Sigma Chemical, Poole, UK). Injections (0.1 μL/min) were made at the stereotaxic coordinates listed in Table 1, using a 5-μL Hamilton syringe (Scientific Glass Engineering, Milton Keynes, UK), adapted with a 34-gauge stainless steel needle. The needle was left in place for 60–120 s after each injection to allow diffusion of the neurotoxin away from the injection site. Following completion of surgery, the skin was sutured and each rat was given postoperative analgesia (Rimadyl®; 4 mg/kg [Carprofen]; Pfizer Ltd, Sandwich, UK). Sham-operated rats received a craniotomy and were then sutured. Rats weighed 315–387 g at the time of surgery and were allowed at least 2 weeks to recover prior to restarting behavioural testing.

**Table 1:** Stereotaxic coordinates for excitotoxic lesions of the OFC and hippocampus, with volumes of toxin injected

	Lesion coordinates			
Lesion	AP	ML	DV	Volume injected (μL)
Orbitofrontal cortex (three sites per side)				
	+4.0	±0.8	−3.4	0.150
	+3.7	±2.0	−3.6	0.200
	+3.2	±2.6	−4.4	0.150
Hippocampus (18 sites per side)				
	−2.4	±1.0	−3.3	0.075
	−2.8	±1.8	−3.3	0.075
	−3.2	±1.4	−3.3	0.050
	−3.2	±1.4	−2.6	0.050
	−3.2	±3.0	−3.1	0.100
	−3.6	±3.5	−3.1	0.075
	−4.4	±2.8	−3.3	0.050
	−4.4	±2.8	−2.3	0.050
	−4.4	±4.0	−4.2	0.025
	−4.4	±4.0	−3.3	0.050
	−4.4	±4.0	−2.3	0.050
	−4.9	±4.8	−5.2	0.075
	−4.9	±4.8	−4.2	0.050
	−5.2	±4.0	−7.3	0.100
	−5.2	±4.0	−4.2	0.075
	−5.2	±4.0	−3.5	0.050
	−5.5	±5.0	−5.6	0.100
	−5.5	±5.0	−4.9	0.075

AP and ML coordinates are in mm relative to bregma. DV coordinates are from the brain surface at the injection site (Paxinos and Watson, 1998) and the skull was level between bregma and lambda.

#### Postoperative testing

Rats were maintained at 85% of their new free-feeding weight. Postoperative testing on the delay task was divided into three phases (phases 2–4; Fig. 4), each consisting of 6 days of testing. Again, each day of testing consisted of two forced trials and five choice trials. The 30 choice trials in each phase were analysed in three blocks of data, consisting of 10 choice trials, from two consecutive days. In phase 2 (the first postsurgical test), the testing procedure was identical to the preoperative testing in phase 1. There was a 10-s delay to reinforcement in the HR arm whereas access to the food reward in the LR arm was immediate (blocks 4–6). In phase 3, an identical 10-s delay was also introduced into the LR arm. Therefore, the rat was now detained for 10 s prior to reinforcement, irrespective of which arm was chosen (blocks 7–9). After phase 3, the original contingencies were reintroduced in phase 4, with again no delay in the LR arm (as in phases 1 and 2). By this stage the majority of rats were choosing the HR arm on the majority of trials, and rarely choosing the LR arm. Because this resulted in little exposure to the change in contingency (there was now no longer a delay present in the LR arm), all rats were first given 2 days of forced trials. They received 10 forced trials per day, five to the HR arm including a 10-s delay, and five to the LR arm with no delay present. Thereafter, the rats received 6 days of testing (two forced and five choice trials) with the original contingencies, a 10-s delay in the HR arm and no delay to reinforcement in the LR arm (phase 4; blocks 10–12).

### Experiment 2: food neophobia

Two tests of food neophobia (hyponeophagia) were next conducted as an additional assessment of the functional effectiveness of the OFC and HPC lesions. These tests rely on the fact that normal animals are less inclined to eat novel foodstuffs in novel, potentially threatening, environments. Previous studies in the laboratory have shown that both HPC-lesioned (Bannerman *et al.*, 2002, 2003; Deacon *et al.*, 2002; McHugh *et al.*, 2004), and OFC-lesioned (Rudebeck *et al.*, 2007) rats are quicker to eat the food in these tests than are controls. For the first test, the rats were presented with pieces of sweetcorn (Green Giant Original Niblets) on an open circular table made from red Perspex (38 cm diameter) and elevated 65 cm above the ground. The sweetcorn pieces were placed in a food well at the centre of the table. Both the testing environment and the sweetcorn were novel to the animal. For the second test, conducted approximately 1 week later, the rats were presented with sucrose Noyes pellets (Sandown Scientific, UK) on one arm of an elevated Y-maze (50 × 9 cm) situated 76 above the floor in another novel testing room. Again, the foodstuff and the testing environment were entirely novel.

For both tests, the rats were food-deprived overnight (for approximately 16 h) prior to testing. The rat was placed in the apparatus facing away from the food. The latencies (i) to make first contact with the food and then (ii) to begin eating were recorded. If the rat had failed to eat within 300 s, it was removed from the apparatus and returned to its home cage for approximately 5 min. It was then retested on two more occasions if necessary. Therefore, if the rat failed to eat within any of these three trials, a maximum latency of 900 s was recorded. The latency to make first contact with the food was subtracted from the latency to eat (latency [eat – contact]) and data were averaged across the two tests.

### Experiment 3: spatial working memory (nonmatching-to-place) testing on the elevated T-maze

#### Apparatus

Spatial nonmatching-to-place (rewarded alternation) testing was conducted on an open, elevated, wooden (unpainted) T-maze in a novel and distinct testing room that was well lit and contained various extramaze cues (benching, posters, cupboards etc.). The T-maze consisted of a start arm (80 cm long and 10 cm wide) and two identical goal arms (60 cm long and 10 cm wide), all arms bordered by 1-cm-high walls. A metal food well (3 cm diameter) was located 3 cm from the end of each goal arm. The maze was elevated 1 m above the floor.

#### Testing

Rats were maintained at 85% of their free-feeding weight and were habituated to this new maze and testing room until they were running freely and consuming the food rewards (45-mg Noyes pellets). Spatial nonmatching-to-place (rewarded alternation) testing then began.

#### Spatial nonmatching-to-place testing

Each trial consisted of a sample run and a choice run. On the sample run the rats were forced either left or right by the presence of a wooden block, according to a pseudorandom sequence (with equal numbers of left and right turns per session, and with no more than two consecutive turns in the same direction). A single pellet reward was available in the food well at the end of the arm. The block was then removed and the animal placed, facing the experimenter, at the end of the start arm and allowed a free choice of either arm. The delay interval between the sample run and the choice run was approximately 10–15 s. The rat was rewarded with two pellets for choosing the previously unvisited arm (i.e. for alternating). If the rat re-entered the arm visited on the sample run, there was no reward available and the animal was immediately removed from the maze. Entry into an arm was defined when a rat placed all four paws into that arm. Animals were run one trial at a time with an intertrial interval (ITI) of approximately 10 min. For this stage of the experiment, the rats received 30 trials in total (five trials/day).

#### Spatial nonmatching-to-place testing with additional delays

Testing then continued for all of the animals but with increasing delays of either 30 or 600 s interposed between the sample run and choice run of each trial. Testing was as in the previous stage of the study, except that during the delay period between sample and choice runs the animals were kept in a separate holding cage (24 × 13 × 13 cm). The rats had previously been well habituated to these holding cages. The rats were returned to their home cages between trials for the ITI (approximately 15 min). Delays were interleaved with each rat receiving 10 trials at each delay condition (20 trials in total). Rats received just two trials per day (one trial with each delay condition) to allow for the contemporaneous testing of all the rats in the experiment.

### Experiment 4: spatial reference memory testing in the Morris water maze

#### Apparatus

Spatial reference memory acquisition was then assessed in an open-field water maze. Rats were trained in the same water maze that we have used previously, which is 2 m in diameter with 60-cm-high walls (Bannerman *et al.*, 2002). The pool was located in a novel and distinct testing room which was well lit and had various prominent extramaze cues (posters, wall cupboards, a medical screen, racks of experimental equipment). The pool was filled with water (25 ± 1°C), and milk was added to obscure the platform and to aid tracking of the animals’ swim paths. In order to escape from the water the rats had to find a hidden escape platform (diameter 10 cm) submerged approximately 1 cm below the water surface, which remained in a fixed location throughout testing. The platform was located at the centre of one of the four quadrants of the pool (arbitrarily designated NE, NW, SE, SW), 50 cm from the sidewall. The number of rats trained to each platform position was counterbalanced with respect to group.

#### Acquisition

Animals had no swim pretraining prior to the start of spatial testing in the water maze. They received four trials per day for eight training sessions (days 1–4 and 6–9), with an ITI of approximately 45 s (of which 30 s was spent sitting on the platform). Rats were placed into the pool facing the side wall at one of eight start locations (nominally N, S, E, W, NE, NW, SE and SW, chosen randomly across trials), and allowed to swim either until they found the platform or for a maximum of 90 s. Any rat that failed to find the platform within the allotted time was guided to its location by the experimenter and allowed to remain on the platform for 30 s before commencing the next trial.

#### Probe trial performance

On the fifth (24 h after spatial training trial 16) and tenth days of testing (24 h after spatial training trial 32), a probe trial was conducted to determine the extent to which the rats had learned about the spatial location of the platform. The platform was removed from the pool and the animals allowed to swim freely for 60 s. For the analysis of the probe trial data, because the fourth quadrant data point was never independent of the other three, the *P*-values were adjusted to reflect a reduction in the degrees of freedom in both the main effect of quadrant and the group × quadrant interaction.

### Experiment 5: spatial reversal learning in the Morris water maze

#### Testing

The performance of the OFC- and sham-lesioned rats on spatial reversal learning was then assessed. The HPC-lesioned rats were substantially impaired during the initial water-maze acquisition and so were not tested further. The remaining rats were first given 4 days (four trials per day) of retraining to the original platform location. Twenty-four hours after the fourth day of retraining, serial spatial reversal testing began.

Testing was conducted as during acquisition with four training trials per day. For the first reversal, the platform was moved to the ‘Opposite’ quadrant for each rat (i.e. 180° away from the ‘Training’ location; see Supporting information, Fig. S1). The radial distance from the centre of the pool remained unchanged (50 cm). Rats received 4 days of training to this new platform position (reversal 1). For reversal 2, the platform was moved by 90° into the ‘Adjacent Right’ quadrant (with respect to the original training quadrant as used during acquisition). In addition, the radial distance between the centre of the pool and the platform was increased to 75 cm (i.e. 25 cm from the inner wall of the pool). The rats then received another 4 days of training to this new platform location. For reversal 3, the platform position was moved by 180° to a point in the ‘Adjacent Left’ quadrant (with respect to the original acquisition training quadrant) that was just 25 cm from the centre of the pool. After 4 days of training to this novel platform location, a final reversal (reversal 4) was conducted with the platform being moved to a point at 135° from the original platform position, at a radial distance of 62.5 cm from the centre of the pool. The rats received just a single day of testing to this final platform location (four trials). No probe trials were conducted during spatial reversal testing.

### Histology

Upon completion of behavioural testing, the rats were terminally anaesthetized with a pentobarbital solution (200 mg/kg by intraperitoneal injection; Euthatal, 200 mg in 1 mL; Vericore Ltd., Dundee, UK) and perfused transcardialy with physiological saline (0.9% NaCl) followed by 10% formalin in 0.9% NaCl. The brain was then removed by dissection and inspected for cortical damage before being stored in 10% formalin. Subsequently the brains were transferred to a 30% sucrose–formalin solution for 24 h, frozen, and then sectioned. Fifty-micrometre horizontal sections were taken from the HPC-lesioned animals and 25-μm coronal sections were taken from the OFC-lesioned animals. A combination of horizontal and coronal sections were taken from the sham-lesioned animals. The sections were mounted on glass slides and stained with Cresyl violet.

## Results

### Histology

**Fig. 2:**
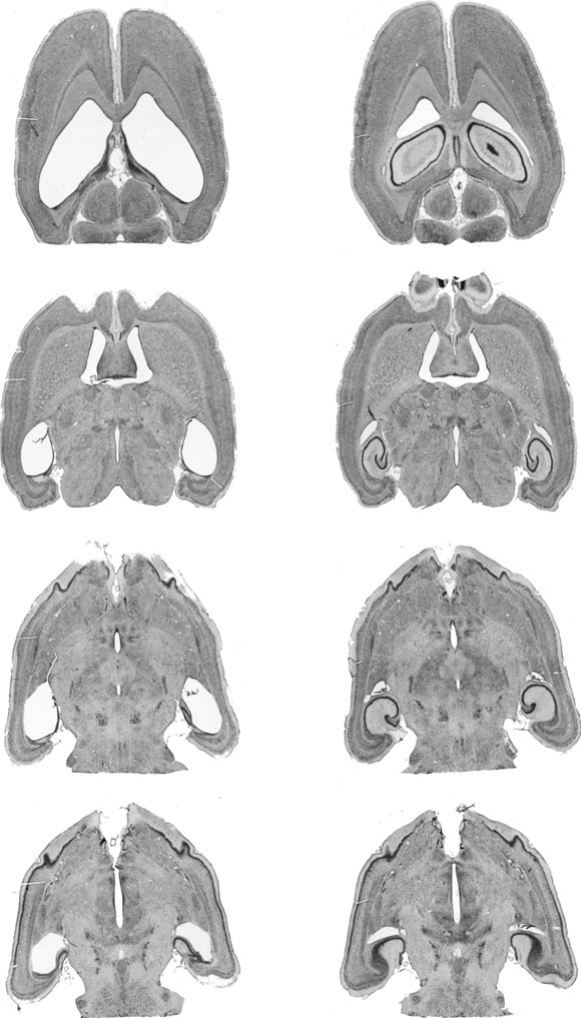
Representative photomicrographs of sham (right) and HPC (left) lesions. Horizontal sections (top to bottom: approximately −3.10, −5.32, −7.34 and −8.42 mm ventral from bregma) showing standard cell loss in a representative HPC-lesioned subject (see also supporting Fig. S2).

Lesions of the hippocampus were highly selective, with near-complete destruction of all of the principal cell layers (Fig. 2; see also supporting Fig. S2). The HPC lesions were very similar to those previously described from this laboratory (Bannerman *et al.*, 1999; McHugh *et al.*, 2008). Cell loss was limited almost exclusively to the hippocampal subfields, with complete loss of pyramidal and granule cells in the dorsal part of Ammon’s horn and the dentate gyrus respectively. However, at the most ventral tip of the hippocampus there was a very small amount of sparing, mainly of the most posterior portion of the dentate gyrus. There was only minimal damage to the subiculum and no visible damage to structures beyond the subiculum such as the amygdala or entorhinal cortex. All rats in the HPC-lesion group were retained in the study for statistical analysis (*n* = 12).

**Fig. 3:**
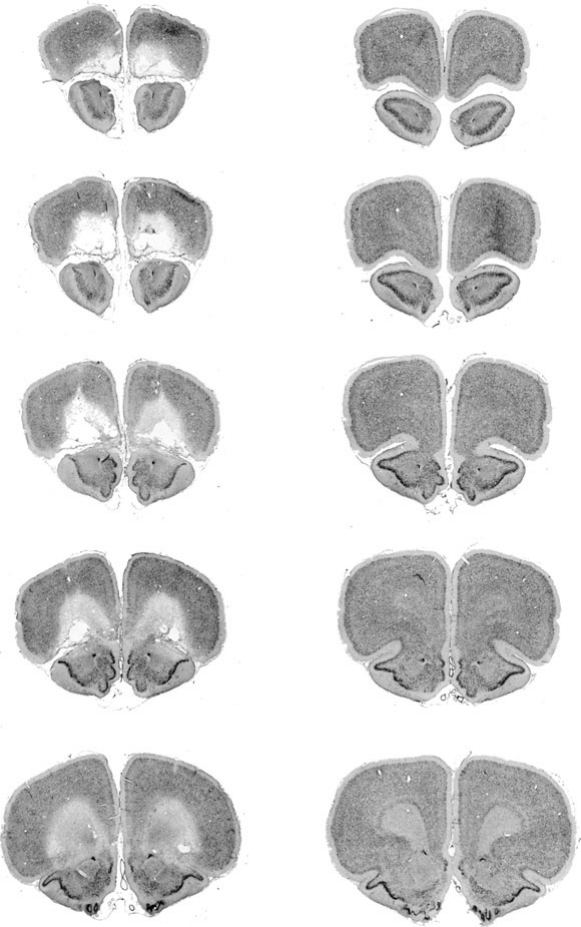
Representative photomicrographs of sham (right) and OFC (left) lesions. Coronal sections (top to bottom: approximately +4.7, +4.2, +3.7, +3.2 and +2.7 mm anterior to bregma) showing standard cell loss in a representative OFC-lesioned subject (see also supporting Fig. S3).

OFC lesions were also highly selective (see Fig. 3; see also supporting Figs S3 and S4), and resembled similar lesions that have been reported previously from this laboratory (Rudebeck *et al.*, 2006, 2007). The lesions included damage to the ventral and lateral areas of the OFC, with varying degrees of sparing to medial and dorsolateral areas. Cell loss commonly extended from the frontal pole (5.2 mm anterior to bregma) to between 2.7 and 3.2 mm anterior to bregma. There was only a minimal amount of damage to the overlying cortex and to the olfactory bulbs beneath. One animal was excluded from the study on the grounds that the lesion was unilateral, leaving a final OFC group size of 11 (unless otherwise stated below). One animal in the sham group died during surgery, leaving a final sham group size of 11 (unless otherwise stated below).

### Experiment 1: nonspatial cued delay cost–benefit decision making

One animal from the sham group developed a middle ear infection and its data were discarded from experiments 1 and 2. The final group sizes in experiment 1 were therefore sham, *n* = 10; OFC, *n* = 11; and HPC, *n* = 12. During preoperative testing (phase 1, blocks 1–3), all rats chose the HR arm on the majority of trials. After surgery (phase 2, blocks 4–6), rats in the HPC lesion group showed an increased preference for the immediate LR arm, whereas both rats in the OFC group and sham-operated controls continued to choose the delayed HR option at roughly the same levels as seen preoperatively (Fig. 4). The results of phases 1 and 2 were included in a three-way anova, with a between-subjects factor of group and within-subject factors of phase and block. This analysis revealed a significant group × phase interaction (*F*_2,30_ = 7.37, *P* < 0.005). There was also a trend towards a significant main effect of group (*F*_2,30_ = 2.59, *P* = 0.09) and a significant main effect of phase (*F*_1,30_ = 16.16, *P* < 0.0005). There was no effect of block or any significant interaction involving block (all *P* > 0.30). Subsequent investigation of the group × phase interaction, using analysis of simple main effects, showed that there was a significant group difference in phase 2 (*F*_2,30_ = 7.82, *P* < 0.005), but not in phase 1 (*F* < 1). Newman–Keuls *post hoc* pairwise comparisons demonstrated that the HPC rats chose the immediate LR arm significantly more often than both the OFC and sham groups (both *P* < 0.01). The sham and OFC groups did not differ (*P* > 0.05). Furthermore, there was a significant effect of phase for the HPC rats (*F*_1,30_ = 10.55, *P* < 0.005), but not for either the sham or OFC groups (both *F* < 1).

In phase 3, a 10-s delay was then also introduced into the LR arm. Now all the animals chose the HR arm on the majority of trials, including the rats with HPC lesions (Fig. 4). A three-way anova including both phases 2 and 3 (blocks 4–9) produced significant main effects of group (*F*_2,30_ = 4.37, *P* < 0.05), phase (*F*_1,30_ = 32.08, *P* < 0.0001) and block (*F*_2,60_ = 5.97, *P* < 0.005). There was also a significant group × phase interaction (*F*_2,30_ = 4.98, *P* < 0.05), and subsequent analysis of simple main effects confirmed that although the three groups differed in phase 2 (see above) they did not differ during phase 3 (*F* < 1). Furthermore, there was a significant effect of phase for the HPC-lesion group (*F*_1,30_ = 12.11, *P* < 0.005), reflecting their increased choice of the HR arm when an equivalent delay was present in both arms.

In phase 4, the original contingencies were reinstated, such that there was a 10-s delay to reinforcement only in the HR arm, and the rats had immediate access to the food in the LR arm. Once again, rats in the HPC group shifted their preference and increased their choice of the immediate LR arm (Fig. 4). A three-way anova comprising the results from phases 3 and 4 showed significant main effects of group (*F*_2,30_ = 4.42, *P* < 0.05) and phase (*F*_1,30_ = 10.94, *P* < 0.005), and significant interactions between group and phase (*F*_2,30_ = 4.45, *P* < 0.05), and between phase and block (*F*_2,60_ = 13.03, *P* < 0.0001). Further exploration of the group × phase interaction using simple main effects revealed a significant difference between groups for phase 4 (*F*_2,30_ = 5.46, *P* < 0.01) and also a significant effect of phase for the HPC-lesion group (*F*_1,30_ = 6.55, *P* < 0.05). Further analysis of the main effect of group in phase 4 using Newman–Keuls *post hoc* pairwise comparisons confirmed that the HPC group chose the immediate LR arm significantly more often than did the sham and OFC groups (both *P* < 0.05), and that the sham and OFC groups themselves did not differ (*P* > 0.05).

Inspection of the data revealed no consistent pattern in the way that individual HPC rats responded when the group as a whole were displaying near-chance levels of performance overall (see supporting Appendix S1). During phase 2, the performance of the individual HPC-lesioned rats varied from 43.3 to 86.7% HR choices across the 30 trials. Furthermore, whereas some HPC-lesioned rats predominantly turned in the same direction on trials on which they chose the LR arm, others showed a very equal distribution of left and right turns when choosing the LR. During phase 4, there was more variation in the number of total HR choices made by the HPC-lesioned rats (ranging from 20 to 100% when averaged across the 30 trials). Interestingly, there was no correlation across the 12 HPC-lesioned subjects between phase 2 and phase 4 in terms of percentage HR choices. The extent and absolute direction of any spatial or response bias also varied from phase 2 to phase 4 for individual subjects. Furthermore, the presence of a directionality bias was not a reliable predictor of impulsive choice in the HPC-lesioned rats (see supporting Appendix S1).

### Experiment 2: food neophobia

**Fig. 5:**
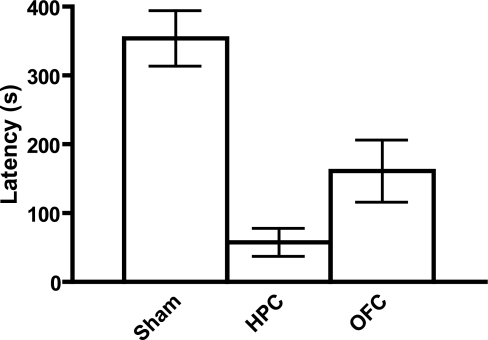
Both HPC and OFC lesions reduced hyponeophagia in the rat. Mean (±SEM) latency [eat – contact] averaged across the two tests of food hyponeophagia.

The final group sizes in experiment 2 were sham, *n* = 10; OFC, *n* = 11; and HPC, *n* = 12. There were no significant differences between groups in terms of the latency to make first contact with the food (*F*_2,30_ = 2.10, *P* > 0.10; see Table 2). Both HPC- and OFC-lesioned rats were faster to begin eating than controls (Table 2). Analysis of the latency [eat – contact] revealed a significant overall main effect of group (*F*_2,30_ = 17.15, *P* < 0.001; Fig. 5). *Post hoc* pairwise comparisons (Newman–Keuls) revealed that both the HPC and OFC groups were significantly quicker to start eating than the sham animals (both *P* < 0.001), and that the two lesion groups were also significantly different (*P* < 0.05), with the HPC rats faster to eat than the OFC rats.

**Table 2:** Experiment 2: food neophobia

	Latencies (s)			Statistics	
Performance measure	Sham (*n* = 10)	HPC (*n* = 12)	OFC (*n* = 11)	*F*_2,30_-value	*P*-value
(i) Contact	14.2 ± 1.7	14.2 ± 1.2	21.8 ± 4.9	2.10	> 0.10
(ii) Eat	368.0 ± 41.0	71.6 ± 20.4	182.8 ± 48.9	15.4	< 0.001
(iii) [Eat – contact]	353.8 ± 40.2	57.4 ± 20.4	161.0 ± 45.2	17.2	< 0.001

Latency values are mean ± SEM, (i) to make first contact with the food, (ii) to begin eating, and (iii) [eat – contact]. Newman–Keuls *post hoc* pairwise comparisons revealed significant differences between sham and HPC (*P* < 0.001), sham and OFC (*P* < 0.005) and HPC and OFC (*P* < 0.05) for latency to eat. There were also significant group differences between sham and HPC (*P* < 0.001), sham and OFC (*P* < 0.001) and HPC and OFC (*P* < 0.05) for latency [eat – contact].

### Experiment 3: spatial working memory (nonmatching-to-place) testing on the elevated T-maze

**Fig. 6:**
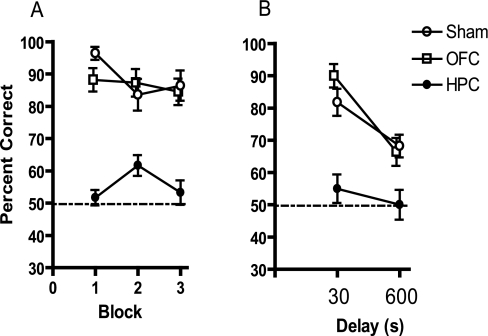
The hippocampus but not the OFC was required for spatial nonmatching-to-place rewarded-alternation performance on the elevated T-maze. (A) Mean percentage correct responses (±SEM) per block of 10 trials for sham-operated (white circles), OFC-lesioned (white squares) and HPC-lesioned (black circles) rats during testing with a minimal (approximately 10–15 s) delay between the sample and choice runs of each trial. (B) Mean percentage correct responses (±SEM) per block of 10 trials with an additional delay of either 30 or 600 s between the sample and choice runs of each trial.

The final group sizes in experiment 3 were sham, *n* = 11; OFC, *n* = 11; and HPC, *n* = 12. Sham- and OFC-lesioned rats displayed equally high levels of performance on the spatial nonmatch-to-place test, alternating between the sample and choice runs on the majority of trials (Fig. 6A). In contrast, rats with HPC lesions displayed near-chance levels of performance (55.6% correct). A two-way repeated-measures anova confirmed that there was a significant between-groups difference (*F*_2,31_ = 66.95, *P* < 0.0001), and *post hoc* pairwise comparions showed that the HPC group was significantly impaired relative to both sham and OFC rats (Newman–Keuls, both at *P* < 0.01), and that these two groups themselves did not differ (*P* > 0.70). There was no main effect of block (*F* < 1), although the group × block interaction did reach significance (*F*_4,62_ = 2.53, *P* < 0.05).

Increasing the length of the delay between the sample run and the choice run reduced the levels of alternation performance in both sham and OFC rats, but did so to an equal extent in both groups (Fig. 6B). The HPC rats continued to display chance levels of performance, irrespective of delay. Anova revealed an overall main effect of group (*F*_2,31_ = 20.59, *P* < 0.0001). *Post hoc* comparisons again confirmed that the HPC group was significantly impaired relative to both of the other groups (both *P* < 0.01), and that the sham and OFC groups did not differ (*P* > 0.70). There was also a main effect of delay condition (*F*_1,31_ = 19.33, *P* < 0.0005) and a trend towards a group × delay interaction (*F*_2,31_ = 2.82, *P* = 0.07).

### Experiment 4: spatial reference memory testing in the Morris water maze

#### Acquisition

The final group sizes in experiment 4 were sham, *n* = 11; OFC, *n* = 11; and HPC, *n* = 12.

**Fig. 7:**
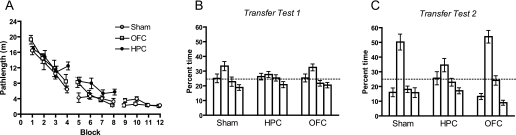
The hippocampus but not the OFC was required for acquisition of the standard spatial reference memory version of the Morris water-maze task. (A) Mean pathlength (±SEM) during acquisition of the reference memory task for sham (white circles), OFC-lesioned (white squares) and HPC-lesioned (black circles) rats. (B) Probe trial 1 after 16 trials; mean percentage time (±SEM) in the adjacent left, training, adjacent right and opposite quadrants (from left to right for each group). (C) Probe trial 2 after 32 trials.

In agreement with previous studies in this laboratory (Bannerman *et al.*, 1999), the HPC-lesioned animals swam faster than either the sham or OFC rats. Mean swim speeds (m/s) during acquisition were: sham, 0.26 ± 0.01; OFC, 0.27 ± 0.01; and HPC, 0.31 ± 0.01; main effect of group, *F*_2,31_ = 19.91, *P* < 0.001; Newman–Keuls *post hoc* comparisons, HPC vs. sham and HPC vs. OFC both at *P* < 0.001 (see supporting Fig. S5). Therefore pathlengths were taken as the measure of performance. All three groups of rats demonstrated a reduction in the distance traveled to find the platform across the 8 days of acquisition training (blocks 1–8; Fig. 7). Rats with HPC lesions showed less improvement and over the 8 days of training were, on average, taking longer paths before finding the platform (sham, 7.9 ± 0.5; OFC, 9.2 ± 0.8; HPC, 10.3 ± 0.7 m; Fig. 7A). An anova conducted on the data from these eight blocks revealed a significant main effect of group (*F*_2,31_ = 3.66, *P* < 0.05) and a main effect of block (*F*_7,217_ = 58.61, *P* < 0.0001). There was a trend towards a group × block interaction (*F*_14,217_ = 1.58, *P* = 0.09). Further analysis of the main effect of group using Newman–Keuls *post hoc* pairwise comparisons showed that the HPC group was significantly impaired relative to the sham-operated animals (*P* < 0.05). A separate comparison of just the sham- and OFC-lesioned animals (excluding the HPC rats) confirmed that there were no differences between these groups (main effect of group, *F*_1,20_ = 2.08, *P* > 0.10; group × block interaction, *F*_7,140_ = 1.02, *P* > 0.40).

#### Probe test performance

During the first probe test (conducted after 16 training trials), the sham and OFC rats displayed a slight preference for the training quadrant whereas the HPC rats searched uniformly across the entire pool (Fig. 7B). However, anova revealed only a significant effect of quadrant (*F*_2,93_ = 8.71, *P* < 0.0001), and no group × quadrant interaction (*F* < 1). There was also no group effect in a one-way anova comparing the time spent in the training quadrant only (*F*_2,31_ = 1.53, *P* > 0.20).

For the second probe test (conducted after 32 training trials) the sham and OFC rats now showed a strong preference for the training quadrant, and this preference was much less pronounced for the HPC group (Fig. 7C). Anova revealed a main effect of quadrant (*F*_2,93_ = 39.54, *P* < 0.0001), and a groups × quadrants interaction (*F*_4,93_ = 3.72, *P* < 0.005). Subsequent analysis of simple main effects revealed that the biggest group difference was for the amount of time spent in the training quadrant (main effect of group for time in the training quadrant, *F*_2,31_ = 4.63, *P* < 0.05). Newman–Keuls *post hoc* comparisons revealed significant differences for training quadrant times between the HPC and OFC groups (*P* < 0.05) and between the HPC and sham groups (*P* < 0.05), but not between the sham and OFC groups (*P* > 0.80).

### Experiment 5: spatial reversal learning in the Morris water maze

#### Retraining

Training continued with just the sham and OFC groups. By now all of the rats were performing well (blocks 9–12; Fig. 7A), and the groups were very well matched for performance during blocks 11 and 12 (*F* < 1, *P* > 0.50 for both). However, analysis of all four retraining blocks revealed that the OFC rats were performing better overall than the sham-operated controls. There was a significant main effect of group (*F*_1,20_ = 5.38, *P* < 0.05), a significant main effect of block (*F*_3,60_ = 5.93, *P* < 0.005) and a significant group × block interaction (*F*_3,60_ = 2.91, *P* < 0.05). Analysis of simple main effects revealed that the OFC rats outperformed the shams on the two training sessions after the second probe test, with a group difference during block 9 (*F*_1,20_ = 5.68, *P* < 0.05) and a marginal effect during block 10 (*F*_1,20_ = 4.26, *P* = 0.05).

#### Spatial reversal testing

**Fig. 8:**
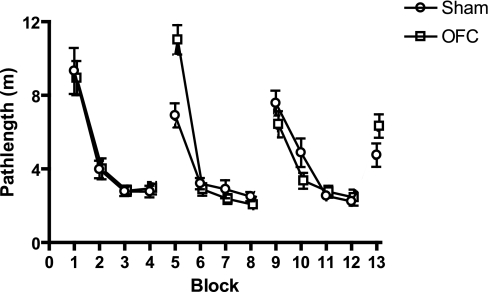
Performance during a series of spatial reversals in the Morris water maze for sham (white circles) and OFC-lesioned rats (white squares). Rats had previously been trained on the standard spatial reference memory fixed-location hidden escape platform task (experiment 4; Fig. 7). They then received four sessions (each consisting of four trials) in which they were trained to a further three novel platform locations. Finally, the rats were given a single test session (four trials) to a fifth different platform location. Mean pathlength (± SEM) during each day of spatial reversal testing.

At this point one rat from the OFC group developed a tumour and was removed from the study. The final group sizes were now sham, *n* = 11; and OFC, *n* = 10. As expected, on the first test block of each spatial reversal (blocks 1, 5, 9 and 13), the rats showed longer paths to the platform, reflecting the fact that they spent some time searching in the previous platform location (Fig. 8). They then improved progressively over the course of the four test blocks to each new platform position. The two groups were well matched in terms of performance during reversal 1. In contrast, the OFC rats were impaired during the first test block of reversal 2 (block 5), taking longer to reach the new platform location.

A two-way repeated-measures anova was performed solely on the first test block of each new reversal stage (i.e. blocks 1, 5, 9 and 13). This examined the extent to which the two groups differed in formation of a reversal learning set, reflecting rapid learning of a new platform location during the first block of each reversal problem. This analysis revealed a significant main effect of block (i.e. reversal stage; *F*_3,57_ = 8.61, *P* < 0.0005), and a significant group × block interaction (*F*_3,57_ = 4.05, *P* < 0.05). There was a trend towards a main effect of group (*F*_1,19_ = 3.51, *P* = 0.08). There was a main effect of trial (*F*_3,57_ = 100.31, *P* < 0.0001) but no significant interactions involving trial (all *F* < 1, *P* > 0.50). Subsequent analysis of simple main effects, exploring the significant group × block interaction, revealed a highly significant group difference on the first block of reversal 2 (*F*_1,19_ = 15.82, *P* < 0.005).

## Discussion

Rats with OFC lesions chose the delayed HR option in preference to the immediate LR option and were indistinguishable from controls when tested on a novel, nonspatial T-maze task in which HR and LR options were associated with patterned goal arms. In contrast, HPC-lesioned rats did display impulsive choice on this task. They also exhibited a substantial spatial learning deficit, both in the water maze and during spatial nonmatching-to-place testing on the elevated T-maze. In contrast, OFC rats displayed normal spatial learning and memory on both tasks. They did, however, exhibit a deficit during the second of four spatial reversals in the water maze, when the platform was moved to a series of novel locations. These results indicate that an intact OFC is not always necessary for intact delay-discounting performance, because rats with OFC lesions were not impaired in choosing the delayed HR option in preference to the immediate LR option in the nonspatial version of the intertemporal choice task. Moreover, the role of the HPC in impulsivity and intertemporal choice is not limited to situations in which the choices leading to delayed HR or immediate LR are between two spatial locations. Thus, the involvement in intertemporal choice of the OFC is more limited, and the involvement of the HPC more extensive, than previously thought.

### HPC and impulsive choice

HPC-lesioned rats displayed impulsive choice on the nonspatial, delay-discounting task. The HPC-lesioned rats did, however, choose the HR option on the majority of trials when there was a 10-s delay associated with both goal arms. When the original contingencies, with no delay in the LR arm, were subsequently reintroduced, the increased preference of the HPC rats for the immediate LR goal arm was then reinstated. Thus, their impairment reflects impulsive choice rather than perseveration to a particular visual cue or direction or movement. Indeed, investigation of the nature of the LR arm choices made by the HPC-lesioned animals showed that the presence of a spatial or response bias was not a reliable predictor of impulsive choice.

The pronounced deficit displayed by the HPC-lesioned rats on the nonspatial ‘cued’ decision-making task (experiment 1) is unlikely to reflect impaired spatial navigation (O’Keefe & Nadel, 1978), and it is well established that hippocampal-lesioned rats are capable of solving simple (gray vs. black-and-white stripes) visual discrimination tasks (Morris *et al.*, 1986; Murray & Ridley, 1999). Therefore, this HPC impairment requires an alternative explanation. It could be that the patterned goal boxes provide contextual cues that become associated with particular reward magnitudes and delays to reinforcement. Hippocampal lesions can disrupt the use of contextual cues to retrieve relevant information (Hirsh, 1974). Importantly, however, the HPC-lesioned rats quickly switched to choosing the HR option on the majority of trials when there was an equal delay present in both the HR and LR goal arms (phase 3; Fig. 4). This suggests that the HPC rats are able to use these ‘contextual cues’ to retrieve information about the size of the reward available in a particular goal arm. Therefore, the data do not support an account based on a general failure of contextual retrieval. The presence of the mediating cue throughout the delay period also argues against a short or intermediate-term memory account (Rawlins, 1985; Schmitt *et al.*, 2004).

One possible explanation for the HPC deficit is that it may reflect a role for the hippocampus in temporal information processing. Studies using the peak interval procedure suggest that HPC lesions lead to an inconsistency in time estimation (Meck *et al.*, 1984; Buhusi & Meck, 2005). Similarly, during testing on the differential reinforcement of low (DRL) rates of responding task, rats with HPC lesions are impulsive and are less able to withhold responding until some minimum time period has elapsed (Bannerman *et al.*, 1999). The present results are consistent with a possible deficit in relative time estimation in HPC-lesioned rats, leading them to overestimate the passing of time. This might explain their impulsive performance on the nonspatial task. Consistent with this possibility, Cheung & Cardinal (2005) have shown that HPC-lesioned rats display impulsive choice on an operant lever-response delay-discounting task, and are sensitive to increasing delays (showing increased preference for the immediate LR choice as delays on the HR option get longer), but with their delay–HR choice function shifted to the left. Furthermore, their performance drops below 50% HR choices (i.e. they demonstrate a significant preference for the LR arm) as delays on the HR get longer. Interestingly, a number of the subjects in our study did show percentage HR choices that were well below 50% during phase 4 (see supporting Table S1). These results are consistent with the possibility that HPC-lesioned rats are less able to encode how recently a stimulus was encountered or an instrumental response was performed, and that relative familiarity may represent a possible mechanism by which these delays are encoded (Fortin *et al.*, 2002; Sanderson *et al.*, 2008).

A further possibility is that the HPC-lesion effect on the present task, at least in part, reflects a reduction in anxiety in these animals (Gray, 1982; Gray & McNaughton, 2000). There is potentially an aversive component to this task, associated with the frustration of the delay to reinforcement in the HR arm. Normal animals might form an association between the aversiveness of the delay period and the larger reward (‘counter-conditioning’). If the HPC-lesioned rats do not perceive the delay as aversive in the same way as controls then this counter-conditioning may not occur. Consistent with this possibility, ventral HPC lesions reduce anxiety and result in impulsive responding on the spatial version of this decision-making task (Bannerman *et al.*, 2002; Kjelstrup *et al.*, 2002; McHugh *et al.*, 2008).

### OFC and impulsive choice

The results of the present study also confirm that OFC-lesioned rats are not always impulsive (see also Winstanley *et al.*, 2004). The absence of an effect of OFC lesions in the present study is in obvious contrast to the pronounced effect of OFC lesions in the previous study of Rudebeck *et al.* (2006), in which the delayed HR and immediate LR were associated with either the left or right goal arms of a uniform gray T-maze. In both studies the animals were trained to a very similar level of performance preoperatively (approximately 85% HR choices). In the Rudebeck *et al.* (2006) study, the OFC-lesioned animals chose the delayed HR arm on < 30% of trials in the immediate postoperative test period. In the present study, the OFC-lesioned rats were indistinguishable from controls, choosing the delayed HR on 80% of trials postoperatively.

Two explanations for the absence of an OFC deficit on the delay-discounting task in the present study can be ruled out. First, the lack of effect was not because the OFC lesions were behaviourally ineffective. OFC-lesioned rats were faster than sham-operated controls to eat novel foods in novel environments (experiment 2, reduced food neophobia; see also Rudebeck *et al.*, 2007). The OFC-lesioned rats also displayed a deficit during the second of four spatial reversals (experiment 5). The OFC lesions were very similar in size and placement to those described for the previous spatial T-maze delay-discounting study, having been generated using the same lesion coordinates and injection volumes (Rudebeck *et al.*, 2006). This is reflected in the similar size of the food neophobia effect for the two cohorts (Rudebeck *et al.*, 2007).

Second, the lack of an effect of OFC lesions on the nonspatial delay-discounting task was not because the task itself was insensitive. A clear impairment was seen in animals with HPC lesions.

One account of the different outcomes from our studies might emphasize the potentially spatial nature of the choices in the task used by Rudebeck *et al.* (2006), in comparison to the visual pattern choices made by the rats in the current experiment. Such an account inevitably suggests a role for the OFC in spatial processes. Although a number of studies have implicated the OFC in spatial learning and memory (Kolb *et al.*, 1983; Corwin *et al.*, 1994; Vafaei & Rashidy-Pour, 2004), the results of experiments 3 and 4 do not support this position; our OFC-lesioned animals were unimpaired on both spatial reference and working memory tasks. This contrast with earlier findings may reflect the use of fiber-sparing neurotoxic lesions in the present study, compared with electrolytic (Corwin *et al.*, 1994) or aspiration (Kolb *et al.*, 1983) lesions, or tetrodotoxin infusions (Vafaei & Rashidy-Pour, 2004) which would disrupt action potentials in fibers of passage through the region of infusion, and could thereby produce effects on spatial learning unrelated to the loss or inactivation of OFC neurons. Thus, the OFC may not be necessary for spatial learning *per se*.

Nevertheless, the different nature of the cues available to the animals in the Rudebeck *et al.* (2006) study and the present study are likely to account for the different outcomes with OFC lesions. However, these cues not only differ in terms of their putative spatial or nonspatial character, but they may also differ in their relative salience. It may be the case that the patterned goal-arm stimuli are more salient than the spatial stimuli used in the previous study. In addition, these visual stimuli are prominent at the choice point, throughout the delay period and when the reward is obtained and consumed.

It has been suggested that the OFC may use information regarding associations between stimuli and particular outcomes, including not only the size of the reward but also the sensory-specific qualities of the reward, to represent expected outcomes and generate predictions (Wise *et al.*, 1996; Schoenbaum *et al.*, 1998, 2003b; Baxter *et al.*, 2000; Schoenbaum & Roesch, 2005; Ostlund & Balleine, 2007; Walton *et al.*, 2007), and that impairments in these processes may account for impulsive choice following OFC lesions (Schoenbaum & Roesch, 2005; Roesch *et al.*, 2007). We suggested previously that, in control animals performing the spatial version of the delay-discounting task (Rudebeck *et al.*, 2006), the OFC may provide a representation of an expected outcome (receiving the HR) that is generated at the choice point and supports the choice of the HR option in preference to the LR option. Without such reward-expectancy signals, OFC-lesioned animals may be less willing to select the delayed option and instead choose the immediately available low reward.

In contrast, in the present study, a willingness to choose the delayed HR option may be maintained in the absence of any representation of specific outcomes, relying instead on a habitual response based on an abstract value of the available reward associated with the stimuli in the goal arms. This in turn could reflect the nature of the stimuli involved, in terms of either their spatial vs. nonspatial features, their salience and/or their prominence throughout the length of each goal arm, from the choice point, through the ‘holding area’ where animals wait on delayed HR trials and finally to the reward. This could be tested directly by using qualitatively different outcomes in the delay-discounting task so that selective satiation manipulations may be utilized to examine the dependence of the choice on the value of the goal; this hypothesis would predict that the spatial version of the task would be sensitive to devaluation but the nonspatial version would not. Importantly, however, the present study shows that the OFC is not always required in order to suppress the choice of an immediate reward over a delayed, larger reward.

### OFC and reversal learning

The OFC-lesioned rats were impaired during the second of four spatial reversals in the water maze, when the platform was moved to a series of novel locations (experiment 5). A number of studies have implicated the OFC in reversal learning, both in primates (Dias *et al.*, 1996; Izquierdo *et al.*, 2004) and in rodents (McAlonan & Brown, 2003; Schoenbaum *et al.*, 2003a). In the present water-maze study, the platform was moved to a series of novel locations with 4 days of training to each new position. Although such testing paradigms have often been described as spatial reversals (Morris *et al.*, 1990), the spatial reversal task in the water maze differs importantly in a number of ways from standard discrimination reversal learning. In this instance the rats are learning a series of new locations in a familiar spatial environment, so that they must avoid returning to a previously rewarded location in favour of a new location rather than alternating between two locations depending on the current reward contingencies. Training rats repeatedly to swim between two familiar locations is less effective in taxing behavioural flexibility because rats find it very easy to learn to return to a previously trained location in the water tank (e.g., Frick *et al.*, 1995).

It has been suggested that reversal learning is slower in OFC-lesioned animals because of the absence of an outcome expectancy, which results in the animals failing to appreciate the change in reward contingencies when the reversal suddenly occurs (Schoenbaum & Roesch, 2005; Schoenbaum *et al.*, 2007). It could be argued that a similar process may be at work in the spatial domain, whereby the OFC is involved in generating a specific representation of the spatial goal (see Feierstein *et al.*, 2006). Of course, such a role for the OFC must by necessity play a limited role in the control of spatial navigation because the OFC-lesioned rats were not impaired in the initial acquisition of the water-maze task, whereas performance was dramatically impaired by HPC lesions. It is possible that, by representing the abstract value of the platform independently of any specific outcome, OFC-lesioned animals are still capable of solving the standard water-maze task. In addition, OFC-lesioned rats were not impaired on the T-maze rewarded-alternation task in which animals have to rapidly and flexibly alter their spatial responses on the basis of trial-specific information provided during the sample run of each trial, although performance on this task may be best explained in terms of simple, nonassociative short-term habituation processes (Sanderson *et al.*, 2008). Normal animals will alternate spontaneously in these maze tasks in the absence of any reward (Sanderson *et al.*, 2007).

Furthermore, although the deficit was statistically robust, the OFC-lesioned rats were only impaired during the initial day of reversal 2, and were indistinguishable from controls on reversals 1 and 3 (there was a nonsignificant trend towards impairment on reversal 4). Therefore this water-maze impairment may not reflect a problem with reversal learning *per se*. Indeed, a general problem with reversal learning might have been expected to result in deficit during the first reversal when the impact of changing the platform position would have been strongest, yet there was no apparent effect of the OFC lesions. Alternatively, the limited OFC-lesion deficit during the reversal phase of the water-maze study may reflect impairment in some other aspect of task performance, such as the acquisition of a learning set or of a particular search strategy. Further studies are required in order to fully understand the contribution of the OFC to performance during serial spatial reversals in the water maze.

## Conclusions

The present study suggests that the impulsivity displayed by OFC-lesioned rats in a number of behavioural paradigms may be best explained in terms of a role for OFC in specific associative learning processes that underlie goal-directed behaviours, and the generation of outcome-specific expectancies. Furthermore, the present study also demonstrated impulsive choice in HPC-lesioned rats in a nonspatial delay-discounting task (experiment 1), a deficit that may be related to impairments in temporal information processing. This result further emphasizes a role for the hippocampus beyond the spatial domain.

## Abbreviations

HPC: hippocampal or hippocampus
HR: high-reward
ITI: intertrial interval
LR: low-reward
OFC: orbitofrontal cortex

## Supporting Information

Fig. S1. Representative platform positions in the watermaze during the initial acquisition phase for an animal trained to the NW quadrant, and then during subsequent spatial reversals.

Fig. S2. Reconstructions of the minimal, representative and maximal HPC lesions.

Fig. S3. Reconstructions of the minimal, representative and maximal OFC lesions.

Fig. S4. Photomicrographs illustrating lesion penumbra in an OFClesioned animal.

Fig. S5. Hippocampal but not OFC lesions increase swim speed during acquisition of the standard, spatial reference memory version of the Morris watermaze task.

Table S1. Spatial response biases in hippocampal lesioned animals.

Appendix S1. Analysis of spatial. response biases in hippocampal lesioned rats during the cost-benefit decision making task.

Please note: As a service to our authors and readers, this journal provides supporting information supplied by the authors. Such materials are peer-reviewed and may be re-organized for online delivery, but are not copy-edited or typeset by Wiley-Blackwell. Technical support issues arising from supporting information (other than missing files) should be addressed to the authors.
