# Supplementary material for: Impulsive choice in hippocampal but not orbitofrontal cortex-lesioned rats on a nonspatial decision-making maze task
Source: Eur J Neurosci. 2009 Aug;30(3):472–84. doi: 10.1111/j.1460-9568.2009.06837.x (PMC2777256; doi:10.1111/j.1460-9568.2009.06837.x)
Supplement: Supplementary file 1 [file ejn0030-0472-SD1.doc]

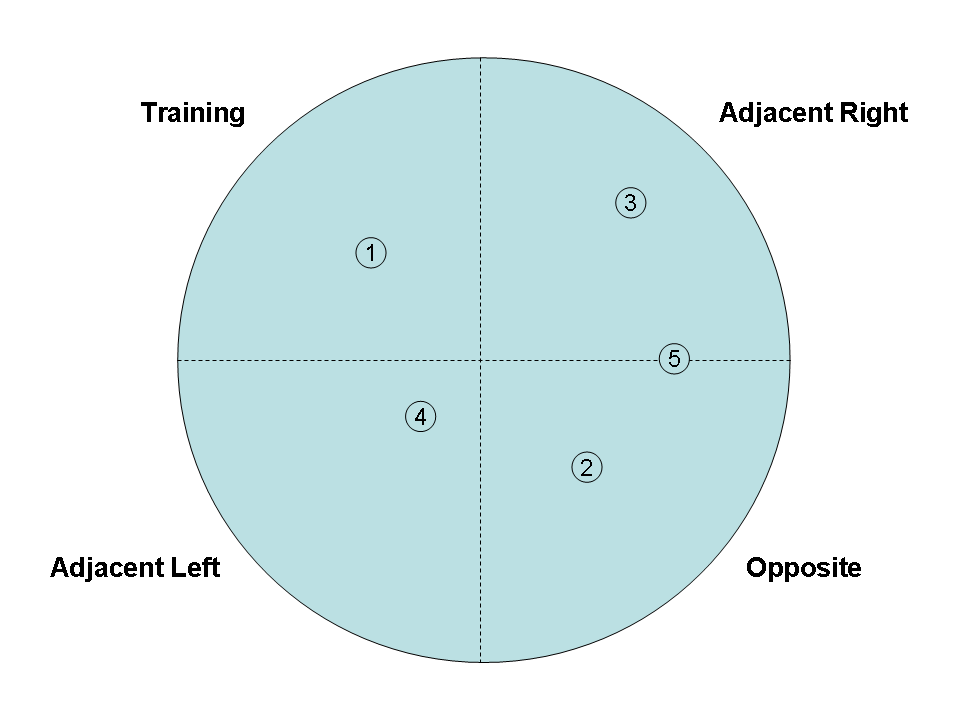


**Fig. S1**. Representative platform positions in the watermaze during the initial acquisition phase (position 1) for an animal trained to the NW quadrant, and then during subsequent spatial reversals (positions 2-5). Four possible platform positions were used during initial acquisition (NW, NE, SW, SE; fully counterbalanced with respect to lesion group), and subsequent positions during reversals were adjusted with respect to the initial training location.
