# Supplementary material for: Impulsive choice in hippocampal but not orbitofrontal cortex-lesioned rats on a nonspatial decision-making maze task
Source: Eur J Neurosci. 2009 Aug;30(3):472–84. doi: 10.1111/j.1460-9568.2009.06837.x (PMC2777256; doi:10.1111/j.1460-9568.2009.06837.x)
Supplement: Supplementary file 2 [file ejn0030-0472-SD2.doc]

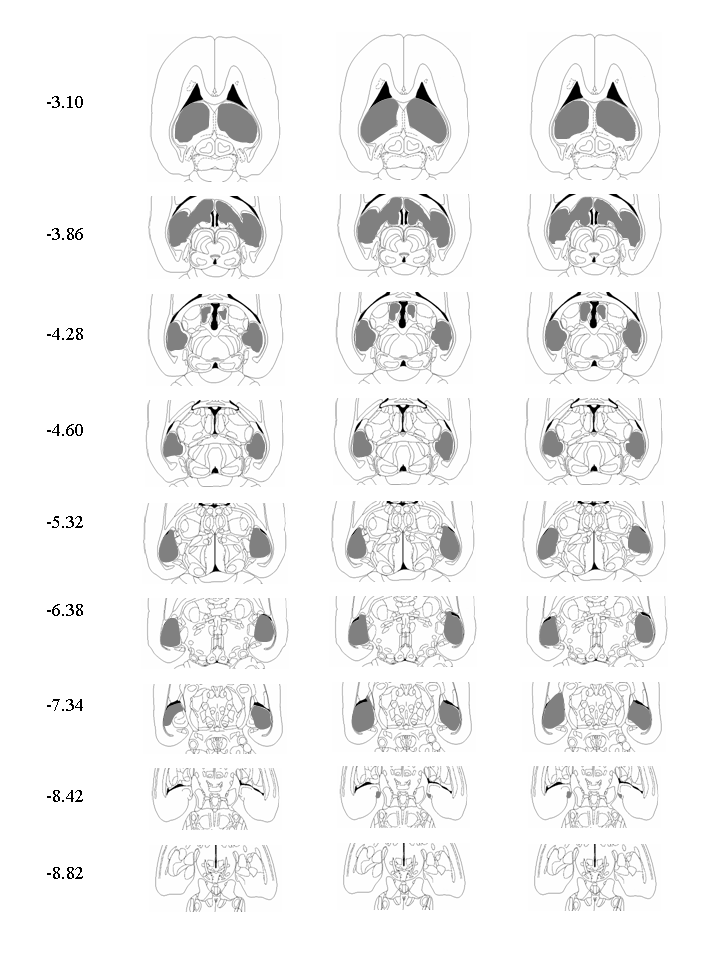


**Fig. S2**. Reconstructions of the minimal (left), representative (centre) and maximal (right) HPC lesions. The size of the lesions in horizontal sections between -3.10 mm and -8.82 mm below bregma are illustrated. Dark shading represents areas of total cell loss.
