# Supplementary material for: Impulsive choice in hippocampal but not orbitofrontal cortex-lesioned rats on a nonspatial decision-making maze task
Source: Eur J Neurosci. 2009 Aug;30(3):472–84. doi: 10.1111/j.1460-9568.2009.06837.x (PMC2777256; doi:10.1111/j.1460-9568.2009.06837.x)
Supplement: Supplementary file 3 [file ejn0030-0472-SD3.doc]

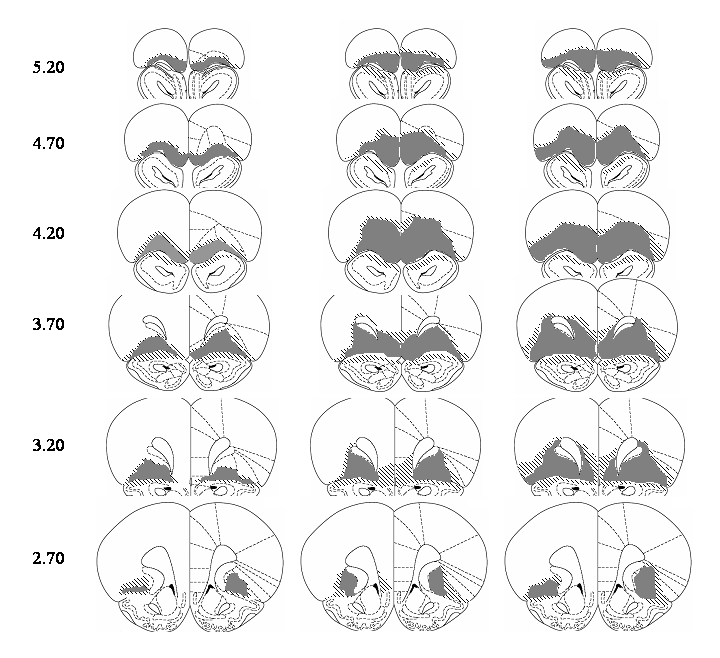


**Fig. S3**. Reconstructions of the minimal (left), representative (centre) and maximal (right) OFC lesions. The size of the lesions in coronal sections between +5.2mm and +2.7 mm anterior to bregma are illustrated. Dark shading represents areas of total cell loss. Diagonal shading represents the lesion prenumba where at least some of the cells were still present but were abnormal compared to those in sham lesion controls (see *Figure S4*).
