# Supplementary material for: Impulsive choice in hippocampal but not orbitofrontal cortex-lesioned rats on a nonspatial decision-making maze task
Source: Eur J Neurosci. 2009 Aug;30(3):472–84. doi: 10.1111/j.1460-9568.2009.06837.x (PMC2777256; doi:10.1111/j.1460-9568.2009.06837.x)
Supplement: Supplementary file 4 [file ejn0030-0472-SD4.doc]

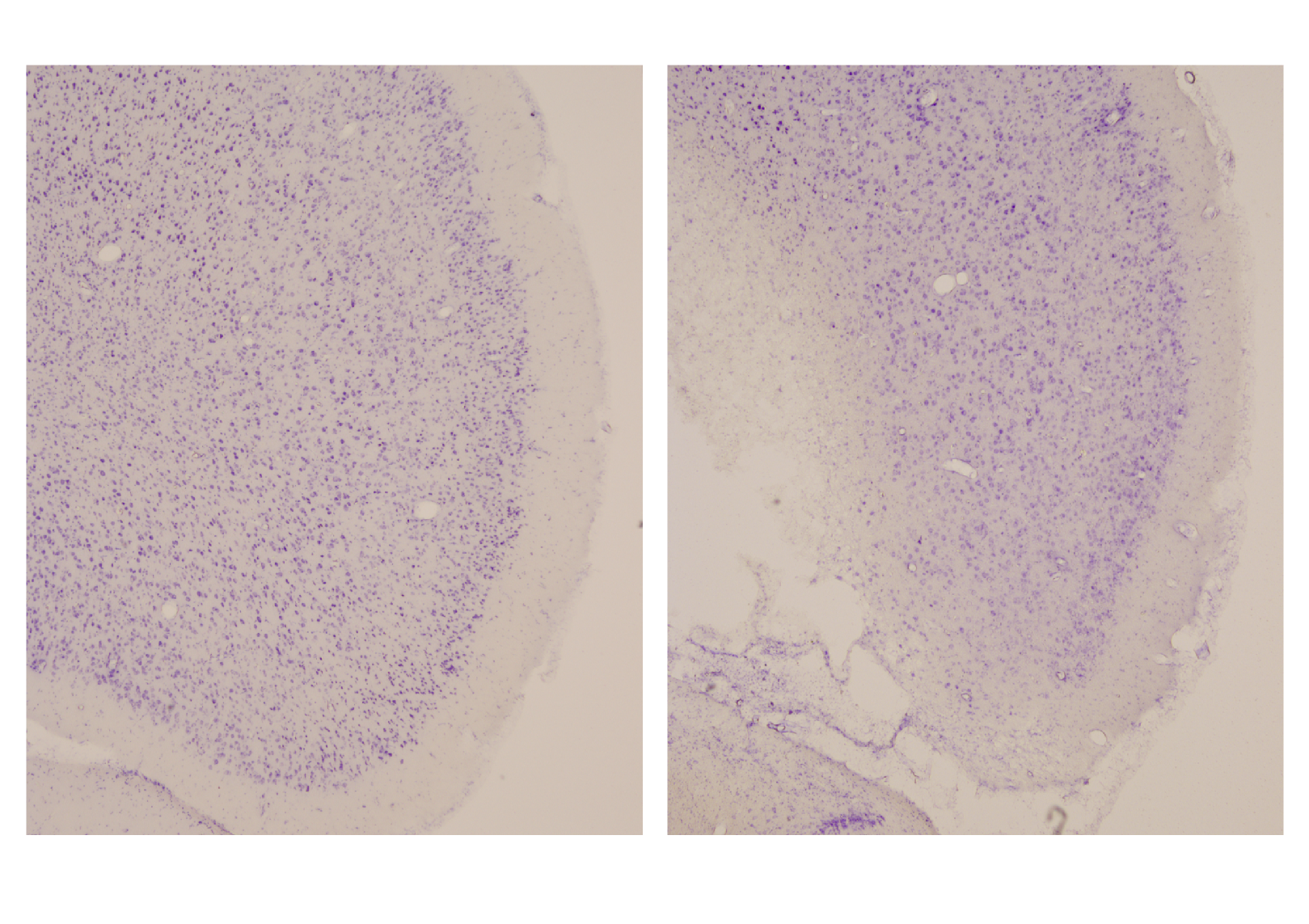


**Fig. S4**. Photomicrographs illustrating lesion penumbra in an OFC-lesioned animal (right). At least some cells are still present in the lesion area but are abnormal compared to those in the sham lesioned controls (left).
