# Supplementary material for: Impulsive choice in hippocampal but not orbitofrontal cortex-lesioned rats on a nonspatial decision-making maze task
Source: Eur J Neurosci. 2009 Aug;30(3):472–84. doi: 10.1111/j.1460-9568.2009.06837.x (PMC2777256; doi:10.1111/j.1460-9568.2009.06837.x)
Supplement: Supplementary file 5 [file ejn0030-0472-SD5.doc]

**Fig. S5**. Hippocampal but not OFC lesions increase swim speed (m/s) during acquisition of the standard, spatial reference memory version of the Morris watermaze task. Mean swim speed (± SEM) during acquisition of the reference memory task for sham (white circles), OFC-lesioned (white squares) and HPC-lesioned rats (black circles). There was a significant main effect of group (F (2,31) = 19.91; p < 0.001) and subsequent Tukey’s HSD pairwise comparisons revealed that the HPC group were significantly different from both the sham and OFC groups (both p < 0.001), but that the OFC and sham groups did not differ (p > 0.20). Furthermore, there were no differences in swim speeds between the OFC and sham groups during either re-training or reversal (both p > 0.20).
