# Supplementary material for: Impulsive choice in hippocampal but not orbitofrontal cortex-lesioned rats on a nonspatial decision-making maze task
Source: Eur J Neurosci. 2009 Aug;30(3):472–84. doi: 10.1111/j.1460-9568.2009.06837.x (PMC2777256; doi:10.1111/j.1460-9568.2009.06837.x)
Supplement: Supplementary file 6 [file ejn0030-0472-SD6.doc]

| **Rat No.** | **Phase 2** | | | | | **Phase 4** | | | | |
| --- | --- | --- | --- | --- | --- | --- | --- | --- | --- | --- |
|  | *% HR choices* | *LR (right)*  *choices* | *LR (left)*  *choices* | *Directionality bias (% of LR choices)* | *Directionality bias (difference score)* | *% HR choices* | *LR (right)*  *choices* | *LR (left)*  *choices* | *Directionality bias (% of LR choices)* | *Directionality bias (difference score)* |
| 4 | 60.0 | 10 | 2 | 83.3 | 8 | 56.7 | 1 | 12 | 92.3 | 11 |
| 7 | 53.3 | 10 | 4 | 71.4 | 6 | 70.0 | 7 | 2 | 77.8 | 5 |
| 10 | 43.3 | 3 | 14 | 82.4 | 11 | 20.0 | 10 | 14 | 58.3 | 4 |
| 13 | 56.7 | 4 | 9 | 69.2 | 5 | 30.0 | 9 | 12 | 57.1 | 3 |
| 15 | 63.3 | 4 | 7 | 63.6 | 3 | 86.7 | 2 | 2 | 50 | 0 |
| 16 | 86.7 | 3 | 1 | 75 | 2 | 60.0 | 5 | 7 | 58.3 | 2 |
| 19 | 63.3 | 6 | 5 | 54.5 | 1 | 50.0 | 2 | 13 | 86.7 | 11 |
| 22 | 60.0 | 7 | 5 | 58.3 | 2 | 66.7 | 1 | 9 | 90 | 8 |
| 25 | 73.3 | 7 | 1 | 87.5 | 6 | 30.0 | 10 | 11 | 52.4 | 1 |
| 26 | 63.3 | 0 | 11 | 100 | 11 | 100* | 0 | 0 | * | * |
| 31 | 56.7 | 11 | 2 | 84.6 | 9 | 26.7 | 10 | 12 | 55.0 | 2 |
| 34 | 80.0 | 1 | 5 | 83.3 | 4 | 100* | 0 | 0 | * | * |

**Table S1.** Spatial response biases in hippocampal lesioned animals during performance on the non-spatial, cued delay cost-benefit decision making task during the immediate post-surgical test period (Phase 2) and during reinstatement of the original training conditions after testing with an equal delay in both goal arms (Phase 4). (* These animals were not included in the correlational analyses for Phase 4).
