# Supplementary material for: Impulsive choice in hippocampal but not orbitofrontal cortex-lesioned rats on a nonspatial decision-making maze task
Source: Eur J Neurosci. 2009 Aug;30(3):472–84. doi: 10.1111/j.1460-9568.2009.06837.x (PMC2777256; doi:10.1111/j.1460-9568.2009.06837.x)
Supplement: Supplementary file 7 [file ejn0030-0472-SD7.doc]

**Appendix S1**

*Analysis of spatial/response biases in hippocampal lesioned rats during the cost-benefit decision making task.*

Hippocampal lesioned animals exhibited impulsive choice on the non-spatial, cued cost-benefit decision making task. They were more inclined to choose the immediate, low reward (LR) option in preference to the delayed, high reward (HR) option compared to sham operated controls. This was true, not only in the test phase immediately post-surgery (Phase 2), but also when the original testing conditions were re-instated after testing with an equal delay in both goal arms (Phase 4; see Figure 4).

To investigate the nature of this deficit we examined the nature of the LR arm choices made by the hippocampal lesioned animals in terms of any putative spatial/response bias (see Supplementary Table). The number of LR arm choices that involved (i) a left turn, and (ii) a right turn were calculated. A directionality bias was then calculated in two ways. First, a percentage directionality bias was calculated. The number of LR arm choices to the dominant side (i.e. the side to which more LR choices were made) was taken and expressed as a percentage of the total number of LR choices (LR choices to the dominant side/(total LR choices)). However, as animals making a small number of total LR choices could show a disproportionately large directionality bias (e.g. a rat making only one LR choice would have a directionality bias of 100%), a second measure of directionality was calculated in which the number of LR choices to the non-dominant side was subtracted from the number made to the dominant side (a difference score). Separate regression analyses were performed to determine whether there was any correlation between the percentage of HR choices and the measures of directionality bias, both for Phase 2 and Phase 4. In Phase 4, two animals chose the HR arm on 100% of trials and so were not included in the correlational analyses

The data are presented in the Supplementary Table. The percentage of HR choices for the hippocampal lesioned animals, collapsed across the 30 trials in Phase 2 varied from 43.3 – 86.7%. Whereas some hippocampal lesioned rats predominantly turned in the same direction on trials in which they chose the LR arm, others showed a very equal distribution of left and right turns when choosing the LR. A regression analysis indicated that there was no correlation between the percentage of HR choices and the degree of any spatial response bias when expressed as a percentage of total LR choices (r (11) = 0.09; p = 0.78). There was, however, a non-significant trend towards a correlation between the directionality bias as a difference score and the percentage of HR choices (r (11) = 0.52; p = 0.08).

During phase 4, there was more variation in the number of total HR choices made by the hippocampal lesioned rats across the group (ranging from 20 – 100% when averaged across the 30 trials). Interestingly, there was no significant correlation between performance in terms of percentage HR choices during Phase 2 and Phase 4 across the 12 hippocampal lesioned subjects (r (11) = 0.41; p = 0.19). Sometimes a spatial/response bias that was present in Phase 2 was not evident in Phase 4 and visa-versa. Indeed, rat 4, for example, actually showed a marked reversal in its spatial/response bias when making LR choices between Phases 2 (right-preferring) and 4 (left-preferring). Furthermore, rat 26 which exhibited a strong spatial/response bias during Phase 2 actually went on to demonstrate 100% HR choices during Phase 4. There were no significant correlations between directionality bias and percent HR choices during Phase 4 for either the percentage (r (9) = 0.33; p = 0.36) or difference score methods (r (9) = 0.12; p = 0.74).
